# Sequence Characterization of Mitochondrial 12S rRNA Gene in Mouse Deer (*Moschiola indica*) for PCR-RFLP Based Species Identification

**DOI:** 10.1155/2013/783925

**Published:** 2013-12-23

**Authors:** Chandra Mohan Siddappa, Mohini Saini, Asit Das, Ramesh Doreswamy, Anil K. Sharma, Praveen K. Gupta

**Affiliations:** ^1^Centre for Wildlife, Indian Veterinary Research Institute, Izzatnagar, Uttar Pradesh 243 122, India; ^2^Department of Veterinary Physiology & Biochemistry, Veterinary College, KVAFSU, Hassan, Karnataka 573201, India; ^3^Division of Animal Biotechnology, Indian Veterinary Research Institute, Izzatnagar, Uttar Pradesh 243 122, India

## Abstract

Mitochondrial 12S rRNA has proven to be a useful molecular marker for better conservation and management of the endangered species. Polymerase chain reaction-restriction fragment length polymorphism (PCR-RFLP) of the mitochondrial 12S rRNA gene has proven to be a reliable and efficient tool for the identification of different Indian deer species of family cervidae. In the present study, mitochondrial 12S rRNA gene sequence of mouse deer (*Moschiola indica*) belonging to the family Tragulidae was characterized and analysed *in silico* for its use in species identification. Genomic DNA was isolated from the hair follicles and mitochondrial 12S rRNA gene was amplified using universal primers. PCR product was cloned and sequenced for the first time. The sequence of mouse deer showed 90.04, 90.08, 90.04, 91.2, 90.04, and 90.08% identities with sika deer, sambar, hog deer, musk deer, chital, and barking deer, respectively. Restriction mapping in Lasergene (DNAstar Inc., Madison, WI, USA) revealed that mouse deer mitochondrial 12S rRNA gene sequence can be differentiated from the other deer species in PCR-RFLP using *Rsa*I, *Dde*I, *Bsr*I, and *Bst*SFI. With the help of predicted pattern, mouse deer can be identified using genomic DNA from a variety of biomaterials, thereby providing molecular aid in wildlife forensics and conservation of the species.

## 1. Introduction

Use of different molecular markers has evolved as a powerful tool in species identification. Earlier, techniques like liquid chromatography [1], immunoassay [2], electrophoresis [3], and so forth were used in species identification. In recent years, use of DNA has been popularized due to its specificity and stability. Among types of DNA [4, 5], mitochondrial DNA has been used extensively due to high copy number of mitochondria in cell. Mitochondria follow clonal inheritance [6] as only mother to contributes to mitochondria; its genome does not undergo recombination; thus, genetic material will be passed onto the next generation unchanged. Reports also suggest that mitochondrial genome is accumulating high percentage of neutral mutations which is helpful in species identification.

Different mitochondrial genes have been used in species differentiation. PCR amplification of cytochrome *b* gene has been used in differentiation of meats from buffalo, emu, and crocodile [7]. Cytochrome *b* has also been used in differentiation of processed products like canned tuna, vegetable oil, and tomato sauce [8]. Other mitochondrial genes like 12S and 16S rRNA are extensively used in mammalian species identification. Numerous molecular techniques have been developed based on the use of mitochondrial 12S rRNA gene identification like RAPD fingerprinting [9], DNA hybridization [10], restriction fragment length polymorphism [11, 12], real time PCR [13], and so forth. Mitochondrial 12S rRNA based PCR- RFLP has been used to differentiate peacock from other poultry species [14] and nilgai from cattle and buffalo [15] as well as differentiation of different deer species belonging to the family Cervidae [16].

Mouse deer also known as chevrotain are small ungulates that belong to the family Tragulidae found in forests of South and Southeast Asia. Indian spotted mouse deer *Moschiola indica* has been recently segregated as a species separately from *Moschiola meminna *[17]. Indian mouse deer, the smallest ungulate in world, lives in solitary or pairs, feeds on plant material, and weighs 1.5 to 18 lbs [18]. Like other ruminants, it has four chambered stomach but third chamber is poorly developed. It is known to evolve from Oligocene 34 million years ago and remained as a primitive ruminant [19]. Mouse deer has been considered as Schedule I animal in Wildlife Protection Act 1972 as its population is declining due to poaching. The poaching is for its skin and meat largely for the pot and often meat is sold in local market (IUCN red list threatened species 2013, http://www.iucnredlist.org/details/136585/0). In the present study, partial sequence of mitochondrial 12S rRNA gene in Indian mouse deer has been characterized for the first time as a tool for species differentiation using PCR-RFLP.

## 2. Materials and Method

### 2.1. Sample

Hairs were collected from Indian spotted mouse deer under captivity at Pilikula Zoo, Mangalore, Karnataka, India, and brought to the laboratory under cold conditions.

### 2.2. Isolation of Genomic DNA

Genomic DNA was isolated from hair follicles using QIAmp tissue extraction kit as per the manufacturer's instructions. The isolated DNA was checked for integrity in 0.8% agarose gel electrophoresis in Tris-acetate EDTA buffer (40 mM Tris-acetate, 2 mM EDTA, pH 8.0) [20].

### 2.3. Amplification of Mitochondrial 12S rRNA Gene

Mitochondrial 12S rRNA gene was amplified using reported [21] universal primers of 12S rRNA, forward primer: 5′ CAA ACT GGG ATT AGA TAC CCC ACT AT 3′ and reverse primer: 5′ GAG GGT GAC GGG CGG TGT GT 3′. Reaction was set up in 50 *μ*L volume consisting of 10X buffer 5.0 *μ*L (10 mM Tris-HCl, 50 mM KCl, and 1.5 mM MgCl_2_), 0.2 mM dNTPs, 20 pmol of each primer, 200 ng template, and 1.5 U proof reading enzyme (Fermentas). Amplification was carried out in a thermal cycler comprising initial denaturation at 94°C for 5 min, followed by 35 cycles of denaturation 94°C for 45 sec, annealing at 59°C for 45 sec, and extension at 72°C for 1.0 min. Final extension was carried out at 72°C for 5 min. The amplicon was separated in 1% agarose gel electrophoresis and the gel spliced product was purified using QIAquick gel extraction kit as per manufacturer's instructions (QIAGEN).

### 2.4. Cloning and Characterization

The purified product was ligated into pJET 2.1 blunt end cloning vector (Invitrogen) using T4 DNA ligase. The ligated product was transformed in to *E. coli* DH5*α* competent cell as per transformation protocol by Chung et al. (1989) and plated onto LB agar with ampicillin (50 *μ*g/mL) as marker. The plate was incubated at 37°C overnight. The obtained colonies were picked into 5 mL LB broth with ampicillin and grown at 37°C overnight under constant shaking at 200 rpm. Plasmids were isolated from overnight grown culture according to standard alkaline miniprep protocol [20].

Recombinant plasmids were screened by PCR as described earlier with T7 universal primer. Plasmids were also subjected to restriction digestion with *Not*1 enzyme. Reaction mixture consisted of 10X buffer O 2 *μ*L, plasmid (1 *μ*g/mL) 5 *μ*L, and *Not*1 2 *μ*L and final volume was made to be 20 *μ*L. Mixture was incubated at 37°C for 16 h and digestion pattern was checked by running 1% agarose gel electrophoresis containing 0.5 *μ*g/mL ethidium bromide. The recombinant plasmid was sequenced at Xcelris Labs Ltd., Anand, Gujarat.

### 2.5. Sequence Analysis

Mitochondrial 12S rRNA gene sequence of Indian mouse deer was analysed with sequences of other deer species like barking deer (AF294731), sambar (M35875), sika deer (AY184433), chital (DQ017832), hog deer (AY775785), and Himalayan musk deer (AY847268) available at NCBI genbank using DNAstar software. Sequence was also used to construct RFLP plot with common restriction enzymes used in other deer species for species identification.

## 3. Results 

**Figure 1:**
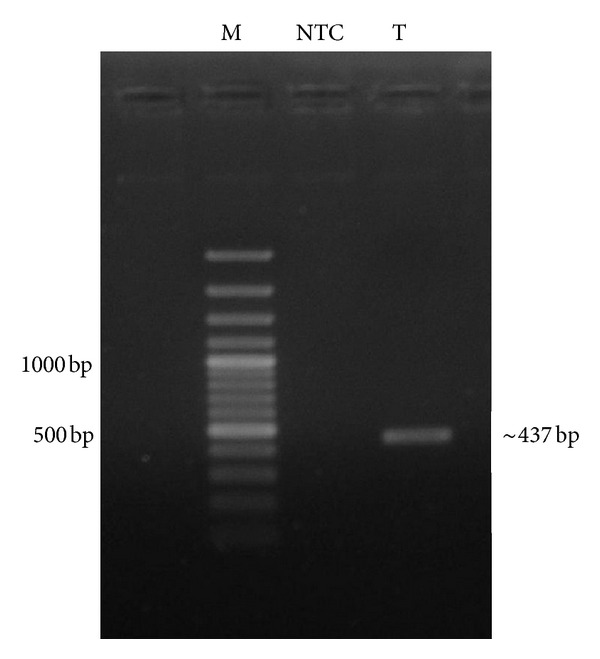
Analysis of the PCR amplicon in 1% agarose gel electrophoresis containing 0.5 *μ*g/mL ethidium bromide. Lane M: 100 bp plus DNA ladder, Lane NTC: no template control, and Lane T: PCR amplicon.

PCR amplification has provided 12S mitochondrial rRNA gene of nearly 437 bp (Figure 1) long nucleotide sequence which was visualized by gel electrophoresis. The recombinant plasmid encoding the amplicon was sequenced which also provided 437 bp sequence. The obtained sequence was blast analysed at NCBI genbank. The sequence submitted to NCBI genbank has been assigned Accession JX570670.1.

Further sequence was analysed *in silico* with other available 12S mitochondrial rRNA sequences deer species with the help of Lasergene (DNAstar Inc., Madison, WI, USA). 

**Figure 2:**
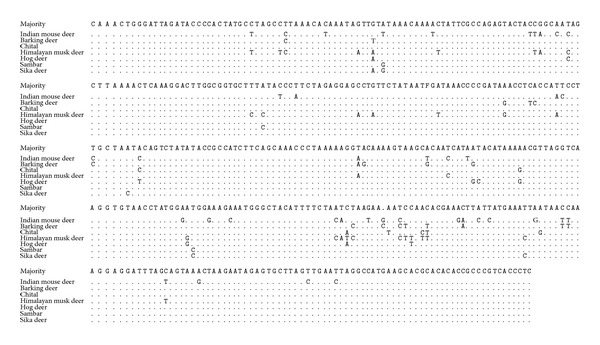
Alignment report of mouse deer 12S mitochondrial rRNA gene sequence with other deer species sequences in Lasergene (DNAstar).

**Figure 3:**
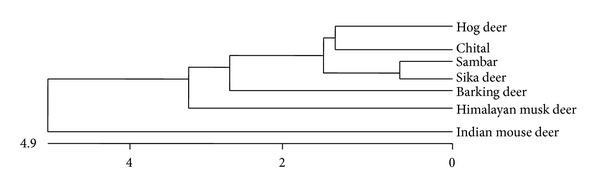
Phylogenetic tree constructed using DNAstar *in silico *software.

Alignment report at nucleotide level showed 90.04, 90.08, 90.04, 91.2, 90.04, and 90.08 percent identity with sika deer, sambar, hog deer, musk deer, chital, and barking deer, respectively. This homology shows close relationship with other deer species (Figure 2). Phylogenetic relationship shows early evolution of mouse deer as separate cluster different from other deer species (Figure 3).

**Figure 4:**
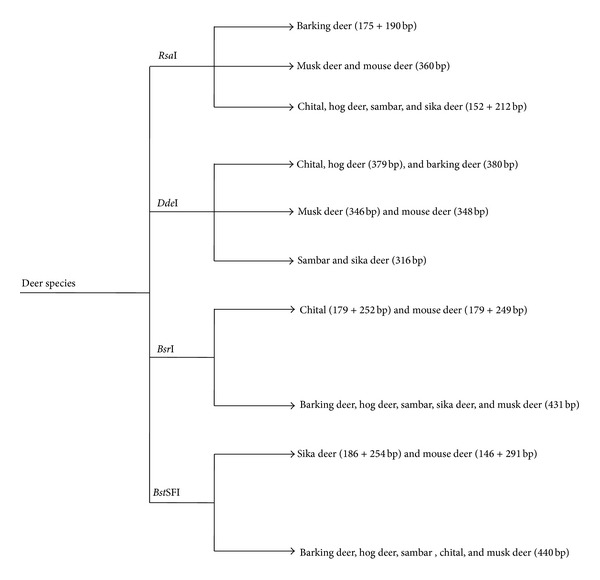
: Restriction fragment length polymorphism (RFLP) of mouse deer and its comparison with other deer species of family Cervidae for species differentiation.

The nucleotide sequence was further analysed with the help of Lasergene (DNAstar Inc., Madison, WI, USA) for restriction mapping which aids in species identification. Fragments predicted in software which cannot be separated in gel electrophoresis have been ignored. Digestion with *Rsa*I, *Dde*I, *Bsr*I, and *Bst*SFI is expected to reveal fragments of 360 bp, 348 bp, 179 + 249 bp, and 146 + 291 bp, respectively (Figure 4 and Table 1).

## 4. Discussion

Many types of molecular markers are used in species identification; among them mitochondrial 12S and 16S rRNA gene have been used extensively due to clonal inheritance of mitochondria without recombination and sequence change. Over a period mitochondrial genome is also accumulating neutral mutations [6], leading to change in restriction enzyme recognition patterns which help in species identification. In the present study mitochondrial 12S rRNA gene was used as molecular marker for differentiation of Indian mouse deer from other deer species. Amplicon comprising ~437 bp was amplified using universal primers which can amplify corresponding region of various organisms including birds and insects [21]. Sequencing and blast analysis of the sequence at NCBI confirmed its identity with other deer species.

Sequence analysis with mitochondrial 12S rRNA gene of other deer species has shown more than 90% similarity which confirms the close relationship with other deer species. Mouse deer belongs to the family Tragulidae, known to evolve very early from Oligocene as primitive ruminant [22]. It is also different from other deer as the third chamber of rumen is poorly developed [23]. From phylogenetic analysis it is evident that it evolved very early as a separate cluster from other deer species family Cervidae (Figure 3). Though nucleotide sequence showed more than 90% homology, the difference in remaining sequence can be used in species differentiation with restriction fragment length polymorphism (RFLP). Restriction digestion with *Rsa*I has given fragment of 360 bp which is similar to musk deer but barking deer (175 + 190 bp) and chital, hog deer, sambar, and sika deer (152 + 212 bp) (Gupta et al., 2008) yield different pattern. Thus, *Rsa*I enzyme can be used to differentiate Indian mouse deer from other deer except musk deer (Figure 4 and Table 1).

Digestion with *Dde*I also yields the same pattern as that of musk deer. The mitochondrial 12S rRNA gene of mouse deer is having two recognition sites for both *Bsr*I and *Bst*SFI which is likely to produce two fragments (179 + 249 bp and 146 + 291 bp resp.,) but musk deer sequence yields only one fragment as it has one recognition site. This can be used to differentiate mouse deer from musk deer (Figure 4 and Table 1). Poaching for the purpose of meat is main threat to Indian mouse deer. With the help of predicted pattern in PCR-RFLP, mouse deer can be identified using genomic DNA isolated from skin or meat samples thereby providing legal molecular aid in wildlife forensics.

## 5. Conclusion 

In the present study nearly 437 bp mitochondrial 12S rRNA gene of Indian mouse deer was amplified. The amplicon was sequenced and analysed *in silico* with other available sequences of deer species. Sequence analysis and restriction mapping in Lasergene (DNAstar Inc., Madison, WI, USA) confirmed the usefulness of 12S rRNA gene as molecular marker for differentiation of mouse deer from other species. Thus, it can be concluded that PCR-RFLP based analysis of mitochondrial 12S rRNA gene can be used as a tool for identification of mouse deer. This will be useful in providing molecular aid in wildlife forensics and conservation of the mouse deer.

## Figures and Tables

**Table 1:** RFLP between different deer species.

Deer species	Amplicon (bp)	*Rsa*I (bp)	*Dde*I (bp)	*Bsr*I (bp)	*Bst*SFI (bp)
Indian mouse deer	437	360	348	179 + 249	146 + 291
Barking deer	440	175 + 190	380	431	440
Chital	440	152 + 212	379	179 + 252	440
Himalayan musk deer	440	360	346	431	440
Hog deer	440	152 + 212	379	431	440
Sambar	440	152 + 212	316	431	440
Sika deer	440	152 + 212	316	431	186 + 254
